# Association between body roundness index and phenoage acceleration among US adults

**DOI:** 10.3389/fpubh.2025.1592274

**Published:** 2025-06-17

**Authors:** Yifei Tan, Ni Zeng, Shiwen Yu, Chaoban Wang, Xintong Jia

**Affiliations:** ^1^Department of Ultrasound, West China Second University Hospital, Sichuan University, Chengdu, China; ^2^Key Laboratory of Birth Defects and Related Diseases of Women and Children, Sichuan University, Ministry of Education, Chengdu, China; ^3^Department of Neonatology, West China Second University Hospital, Sichuan University, Chengdu, China; ^4^Department of Pediatric Hematology, West China Second University Hospital, Sichuan University, Chengdu, China; ^5^Department of Pediatric Gastroenterology, West China Second University Hospital, Sichuan University, Chengdu, China

**Keywords:** Phenoage, phenoage acceleration, body roundness index, BMI, NHANES

## Abstract

**Background:**

Phenoage, compared to chronological age, better captures the multifaceted nature of aging as a process influenced by various pathological and environmental factors. Phenoage acceleration, defined as the disparity between biological and chronological age, indicates aging pace. However, the association between body roundness index (BRI), a more precise measure of obesity, and phenoage acceleration remains unexamined.

**Methods:**

Adults participants were enrolled from the United States National Health and Nutrition Examination Survey (NHANES) conducted between 2005 and 2020. The values of BRI, as well as body mass index (BMI) and phenoage acceleration were calculated. Potential correlations between phenoage acceleration and the values of BRI were explored using multivariate regression models.

**Results:**

Data from 8,848 individuals were analyzed, of which 4,271 (48.3%) participants being female. The overall mean BRI among participants was 5.43 (±2.37), while the mean phenoage was 45.5 (±18.5) years. After full covariate adjustment, a stepwise increase in phenoage acceleration was observed across BRI quartiles. Compared to Quartile 1 (Q1, reference), the acceleration was significantly greater in Q2 (*β* = 1.68, 95% CI: 1.25–2.11), Q3 (*β* = 3.65, 95% CI: 3.20–4.09), and Q4 (*β* = 6.64, 95% CI: 6.18–7.10), indicating a progressively faster aging rate with higher BRI levels. Across all subgroups, higher BRI values were consistently associated with an increase in phenoage acceleration.

**Conclusion:**

Our study reveals a significant positive link between BRI and phenoage acceleration, highlighting BRI’s potential as a sensitive predictor of biological aging. Integrating BRI into routine assessments could enable more personalized and effective strategies for healthy aging.

## Introduction

Chronological age is often perceived as a simple numerical value that increases over time. However, a growing number of consensus (1–3) suggest that aging is better defined by biological markers rather than time alone, as they more accurately reflect the aging process and associated risks of mortality or disease. To this end, the concept of ‘phenoage’ has been proposed in recent years (4). Phenoage, calculated based on DNA methylation (DNAm), represents a composite measure of ‘healthspan’ that integrates multisystem and multifactorial biological influences (4). This approach aligns with the understanding that aging is a multifaceted process influenced by various pathological and environmental factors, rather than being solely a function of time. Thus, phenoage serves as a more precise, comprehensive, and clinically relevant predictor for a range of aging outcomes, including all-cause mortality, cancer, healthspan, and response to interventions (4–6). This framework also introduces the concept of phenoage acceleration, which refers to the difference between biological age (estimated using the phenoage algorithm) and chronological age. A higher value indicates a faster rate of aging. Accurate assessment of phenoage acceleration is essential for a better understand of aging mechanisms and for implementing early interventions. It also aids in evaluating the impact of lifestyle and treatments on biological aging, ultimately contributing improved healthspan and lifespan.

In addition, obesity is a significant contributor to the growing pressure on global healthcare systems. Moreover, studies (7–9) have shown that obesity often accelerates the aging process through metabolic and other pathways. Traditionally, Body Mass Index (BMI; calculated as weight in kilograms divided by height in meters squared) has been widely used as a convenient and easily obtainable indicator of obesity. However, as research into obesity deepens, it has become evident that the complexity of obesity cannot be fully captured by height and weight alone, rendering BMI a relatively simplistic measure. Further researches (10, 11) indicate that body fat distribution, particularly an increase in abdominal fat, is closely associated with aging, potentially through mechanisms such as insulin resistance. Fortunately, the introduction of the Body Roundness Index (BRI) has provided a more comprehensive solution. Although the concept of BRI has been proposed only in recent years, studies have already identified its association with various conditions, including colon cancer risk (12), metabolic syndrome (13), hypertension (14), and others (15). Moreover, research by Zhang X. et al. has demonstrated that BRI is more sensitive indicator of all-cause mortality risk compared to BMI (16). The BRI, a newer metric, builds upon the strengths of BMI while incorporating additional factors such as abdominal fat, thereby providing a more accurate reflection of obesity (17).

Given that obesity assessed by BMI is associated with accelerated aging, the relationship between BRI-a novel index that better reflects body fat distribution-and biological aging is more deserving of investigation. However, to the best of our knowledge, no studies have yet examined the relationship between BRI and phenoage. To fill this gap, we analyzed the association between BRI and phenoage using a nationally representative sample from the United States collected between 2005 and 2020.

## Methods

The National Health and Nutrition Examination Survey (NHANES) is an annual study on the health status of the U. S. population, approved by the Institutional Review Board of the National Center for Health Statistics. All participants provided written informed consent. Since all anonymized data are publicly available through the online platform, this cohort study does not require additional ethical review or informed consent.

### Study participants

**Figure 1:**
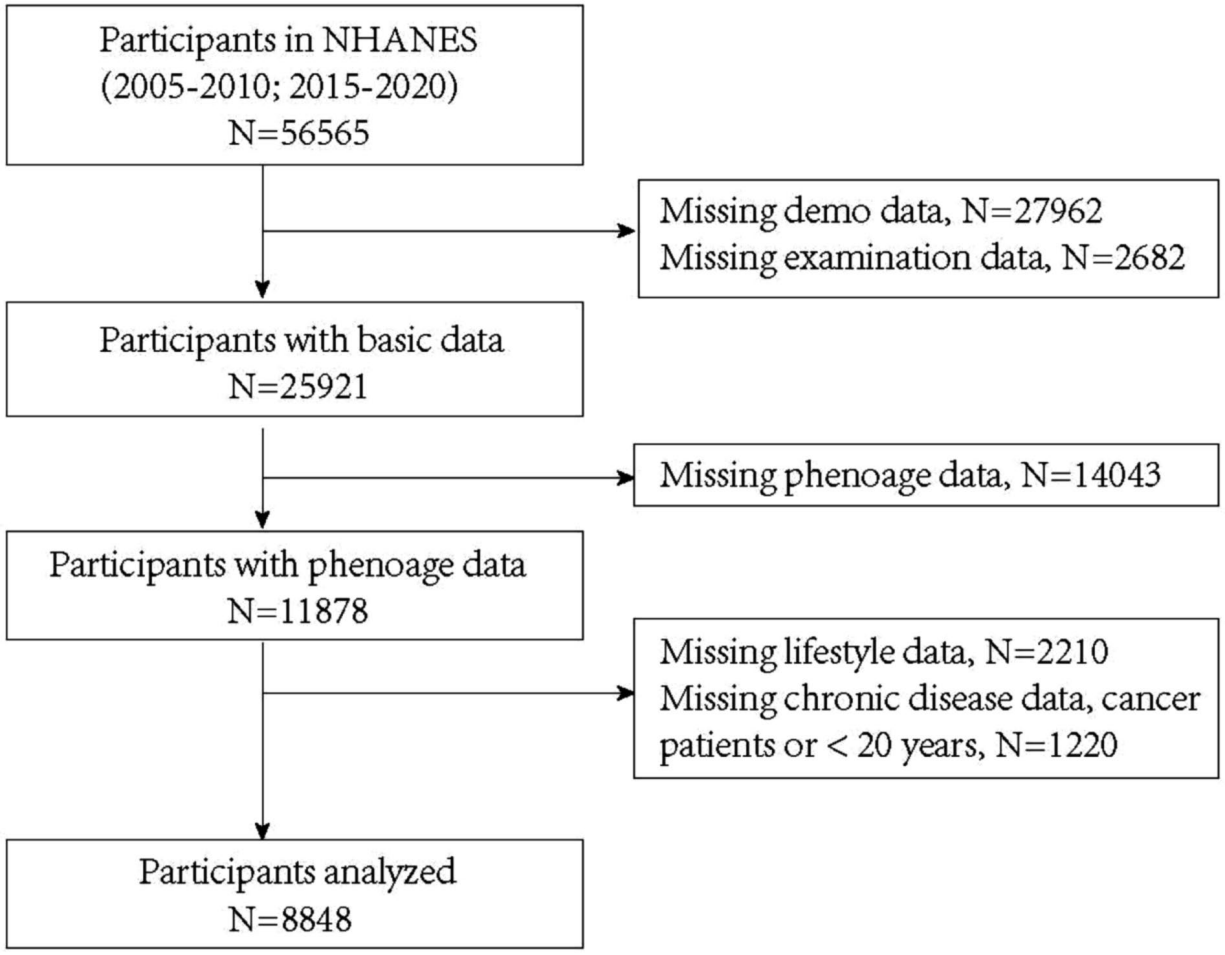
Flowchart of participants selection.

For this analysis, we included participants with C-reactive protein (CRP) data from five NHANES cycles between 2005 and 2020 (2005–2006, 2007–2008, 2009–2010, 2015–2016, and 2017–2020). Participants lacking key clinical data or with a history of cancer were excluded. Among the 56,565 respondents initially eligible for analysis, 30,644 were excluded due to invalid or missing demographic or anthropometric data, 14,043 due to incomplete phenoage data, 2,210 due to missing lifestyle data, and 1,220 due to missing chronic diseases data, a reported history of cancer or being under 20 years old. Ultimately, 8,848 participants were included in the final analysis (Figure 1).

### BRI definition

BRI is calculated based on height and waist circumference, offering a more accurate estimation of body fat percentage and health risks associated with obesity. The formula for BRI is expressed as: 364.2–365.5 × √ (1 − [waist circumference (cm)/2π]^2^ / [0.5 × height (cm)]^2^) (17). BMI is calculated by dividing body weight (kg) by the square of height (m). A BMI between 25 and 29.9 is classified as overweight, while BMI of 30 or higher is classified as obesity.

### Ascertainment of phenoage

Phenoage is a biomarker-based measure of biological aging. The formula for phenoage is derived from a combination of nine biomarkers, including white blood cell count (WBC), albumin (ALB), glucose (GLU), CRP, lymphocyte percentage (LYMPH%), mean corpuscular volume (MCV), red cell distribution width (RDW), alkaline phosphatase (ALP), and creatinine (CREAT), in addition to chronological age. These biomarkers are weighted based on their association with mortality risk (4). Additionally, phenoage acceleration is a metric that quantifies the discrepancy between chronological age and biological age as estimated by the phenoage algorithm.

### Covariates

Information on age, sex, race, educational attainment, family income, smoking status, alcohol drinking, physical activity status and history of cardiovascular disease (CVD), hypertension and diabetes was collected through in-home interviews. Family income was assessed by Poverty income ratio (PIR), which represents the ratio of a family’s income to the poverty threshold, with higher values indicating a higher level of income relative to the poverty line. Race was self-reported by participants using predefined categories, including Mexican American, Other Hispanic, non-Hispanic Black, non-Hispanic White, and Other Race. Educational attainment was classified into the following categories: less than 9th grade, 9th to 11th grade, high school graduate, some college, and college graduate or higher.

### Statistical analysis

Participants were categorized into four quartiles based on their BRI values, and baseline characteristics across the quartiles were compared. Continuous variables are presented as means ± standard deviations, while categorical variables are reported as frequencies (percentages). The differences were assessed using analysis of variance (ANOVA) for continuous data and chi-square tests for categorical data. The potential association between BRI and phenoage was evaluated by constructing restricted cubic splines (RCS) curves, followed by a logistic regression model to examine the dose-dependent relationship between BRI and phenoage. Three regression models were employed: Model 1 included BRI only (unadjusted), Model 2 adjusted for age, sex, race, income, and education, while Model 3 further adjusted for lifestyle factors (smoking, alcohol consumption, and physical activity) and comorbidities (diabetes, hypertension, and cardiovascular disease). Additionally, subgroup analyses were conducted to explore whether the association between BRI and phenoage varied across different populations. Statistical analyses were performed using R software (version 4.3.1), and a two-tailed *p*-value of less than 0.05 was considered statistically significant.

## Results

### Baseline characteristics of participants

After combining data from all included NHANES cycles, the baseline characteristics of the study participants are presented in Table 1. Among 8,848 eligible adults, the mean (SD) age was 45.6 (15.9) years, with 4,271 (48.3%) participants being female. For sensitivity assessment, we divided participants into quartiles based on BRI (Q1: <3.78; Q2: 3.78–5.01; Q3: 5.01–6.64; Q4: ≥6.64). The overall mean BRI among participants was 5.43 (±2.37), while the mean phenoage was 45.5 (±18.5) years. Additionally, as BRI increased, phenoage also progressively rose, indicating that participants with higher BRI tended to be biologically older. Notably, across the BRI quartiles (Q1 to Q4), phenoage acceleration transitions from being less than zero to greater than zero, showing an overall increasing trend.

**Table 1:** Baseline characteristics of participants according to quartiles of body roundness index.

Variables	Total	Q1 <3.78	Q2 3.78–5.01	Q3 5.01–6.64	Q4 >6.64	*p*
Age	45.6 ± 15.9	38.2 ± 14.6	46.2 ± 15.4	48.9 ± 15.5	49.2 ± 15.7	<0.0001
Sex						<0.0001
Male	4,177 (47.2)	1,136 (53.8)	1,186 (56.2)	1,082 (51.2)	773 (36.6)	
Female	4,271 (48.3)	976 (46.2)	926 (43.8)	1,030 (48.8)	1,339 (63.4)	
Race						<0.0001
Mexican American	1,464 (16.5)	202 (9.6)	372 (17.6)	486 (23)	404 (19.1)	
Other Hispanic	848 (9.6)	149 (7.1)	220 (10.4)	247 (11.7)	232 (11)	
Non-Hispanic White	3,419 (38.6)	933 (44.2)	862 (40.8)	788 (37.3)	836 (39.6)	
Non-Hispanic Black	1810 (20.5)	503 (23.8)	393 (18.6)	388 (18.4)	526 (24.9)	
Other race	907 (10.3)	325 (15.4)	265 (12.5)	203 (9.6)	114 (5.4)	
Education						0.0005
Less than 9th grade	745 (8.4)	74 (3.5)	191 (9)	255 (12.1)	225 (10.7)	
9–11th grade	1,090 (12.3)	256 (12.1)	255 (12.1)	282 (13.4)	297 (14.1)	
High school grad/GED	1942 (21.9)	471 (22.3)	456 (21.6)	506 (24)	509 (24.1)	
Some college/AA degree	2,610 (29.5)	644 (30.5)	614 (29.1)	618 (29.3)	734 (34.8)	
College graduate or above	2058 (23.3)	667 (31.6)	596 (28.2)	451 (21.4)	347 (16.4)	
PIR						<0.0001
<1.5	2,869 (32.4)	651 (30.8)	677 (32.1)	720 (34.1)	821 (38.9)	
≥1.5	5,579 (63.1)	1,461 (69.2)	1,435 (67.9)	1,392 (65.9)	1,291 (61.1)	
BMI	29.3 ± 7.0	22.4 ± 2.5	26.5 ± 2.5	30.1 ± 2.9	38.1 ± 6.3	<0.0001
<25	2,425 (27.4)	1771 (83.9)	599 (28.4)	54 (2.6)	1 (0)	
25–30	2,789 (31.5)	339 (16.1)	1,343 (63.6)	1,008 (47.7)	99 (4.7)	
≥30	3,234 (36.6)	2 (0.1)	170 (8)	1,050 (49.7)	2012 (95.3)	
BRI	5.43 ± 2.37	2.91 ± 0.57	4.4 ± 0.36	5.74 ± 0.46	8.68 ± 1.94	<0.0001
Smoking						<0.0001
Never	2,425 (28.7)	1,165 (55.2)	1,211 (57.3)	1,176 (55.7)	1,173 (55.5)	
Former	2,789 (33)	340 (16.1)	447 (21.2)	550 (26)	540 (25.6)	
Current	3,234 (38.3)	607 (28.7)	454 (21.5)	386 (18.3)	399 (18.9)	
Alcohol						<0.0001
None	1,619 (19.2)	314 (14.9)	361 (17.1)	443 (21)	501 (23.7)	
Light	4,131 (48.9)	1,067 (50.5)	1,068 (50.6)	987 (46.7)	1,009 (47.8)	
Moderate	1,579 (18.7)	442 (20.9)	403 (19.1)	405 (19.2)	329 (15.6)	
Heavy	1,119 (13.2)	289 (13.7)	280 (13.3)	277 (13.1)	273 (12.9)	
Moderate physical activity						0.002
Yes	3,849 (45.6)	1,031 (48.8)	944 (44.7)	924 (43.8)	931 (44.1)	
No	4,599 (54.4)	1,081 (51.2)	1,168 (55.3)	1,188 (56.3)	1,181 (55.9)	
Diabetes						<0.0001
Yes	918 (10.9)	53 (2.5)	168 (8)	240 (11.4)	457 (21.6)	
No	7,362 (87.1)	2042 (96.7)	1900 (90)	1827 (86.5)	1,593 (75.4)	
Borderline	168 (2)	17 (0.8)	44 (2.1)	45 (2.1)	62 (2.9)	
Hypertension						<0.0001
Yes	2,648 (31.3)	288 (13.6)	570 (27)	757 (35.8)	1,033 (48.9)	
No	5,800 (68.7)	1824 (86.4)	1,542 (73)	1,355 (64.2)	1,079 (51.1)	
CVD						<0.0001
Yes	334 (4)	33 (1.6)	73 (3.5)	87 (4.1)	141 (6.7)	
No	8,114 (96)	2079 (98.4)	2039 (96.5)	2025 (95.9)	1971 (93.3)	
Phenoage	45.5 ± 18.5	34.8 ± 16.5	44.4 ± 17.1	49.2 ± 17.2	53.5 ± 18	<0.0001
Phenoage acceleration	−0.16 ± 8.15	−3.4 ± 6.52	−1.8 ± 6.92	0.3 ± 7.92	4.28 ± 8.91	<0.0001

BMI, body mass index; CVD, cardiovascular disease; PIR, poverty income ratio.

### Associations between BRI and phenoage

**Figure 2:**
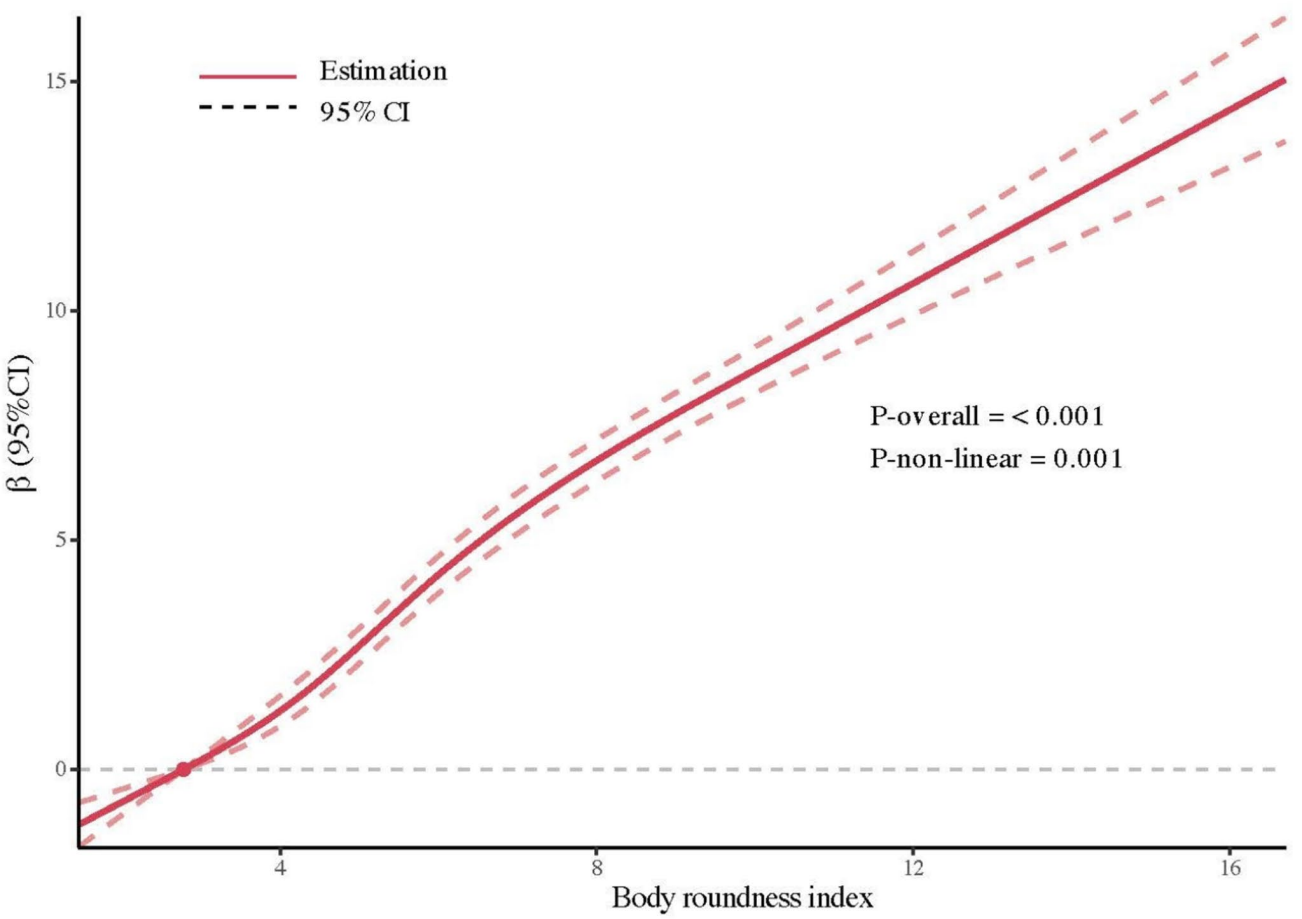
RCS analysis of the association between BRI and phenoage with full adjustments.

Since there are currently no recommended cutoff points for BRI, RCS curves were plotted to illustrate its potential association with phenoage. The results of the RCS analysis (Figure 2) demonstrated a positive overall trend (overall *p*-value < 0.001) and a nonlinear association (nonlinear *p*-value = 0.001) between BRI and phenoage after full adjustments.

Subsequently, multiple variates logistic regression models were employed to further analyze the correlation between BRI and phenoage acceleration, with Q1 of BRI designated as the reference group. Table 2 summarizes the associations between different BRI categories and phenoage acceleration before and after adjusting for sociodemographic factors. In line with the RCS curves, a gradual increase in phenoage acceleration was observed from the BRI Q1 quartile to the Q4 quartile before adjusting covariates and after adjustments for demographic and body examination factors. Following full covariate adjustment, a stepwise increase in phenotypic age acceleration was observed across BRI quartiles. Compared to Q1 (reference), the magnitude of acceleration was significantly higher in Q2 (*β* = 1.68, 95% CI: 1.25–2.11), Q3 (*β* = 3.65, 95% CI: 3.20–4.09), and Q4 (*β* = 6.64, 95% CI: 6.18–7.10), suggesting enhanced aging velocity with increasing BRI levels.

**Table 2:** Association between body roundness index and phenoage acceleration according to multivariate regression models.

Without adjustment	*β* (95% CI)	*p*
Q1 (<3.78)	Reference	
Q2 (3.78–5.01)	1.60 (2.06–1.14)	<0.0001
Q3 (5.01–6.64)	3.70 (4.16–3.24)	<0.0001
Q4 (>6.64)	7.68 (8.14–7.22)	<0.0001
Adjusted for demo information		
Q1 (<3.78)	Reference	
Q2 (3.78–5.01)	1.68 (2.14–1.23)	<0.0001
Q3 (5.01–6.64)	3.81 (4.28–3.35)	<0.0001
Q4 (>6.64)	7.78 (8.26–7.31)	<0.0001
With full adjustments		
Q1 (<3.78)	Reference	
Q2 (3.78–5.01)	1.68 (2.11–1.25)	<0.0001
Q3 (5.01–6.64)	3.65 (4.09–3.20)	<0.0001
Q4 (>6.64)	6.64 (7.10–6.18)	<0.0001

In a similar manner, we also analyzed the association between BMI and phenoage acceleration. While BMI was also positively correlated with phenoage acceleration (Supplementary Table S1; Supplementary Figure S1), BRI demonstrated greater sensitivity in capturing this association, as evidenced by the higher *β* observed across the same quartiles and lower *p* for nonlinear.

### Subgroup analyses

**Figure 3:**
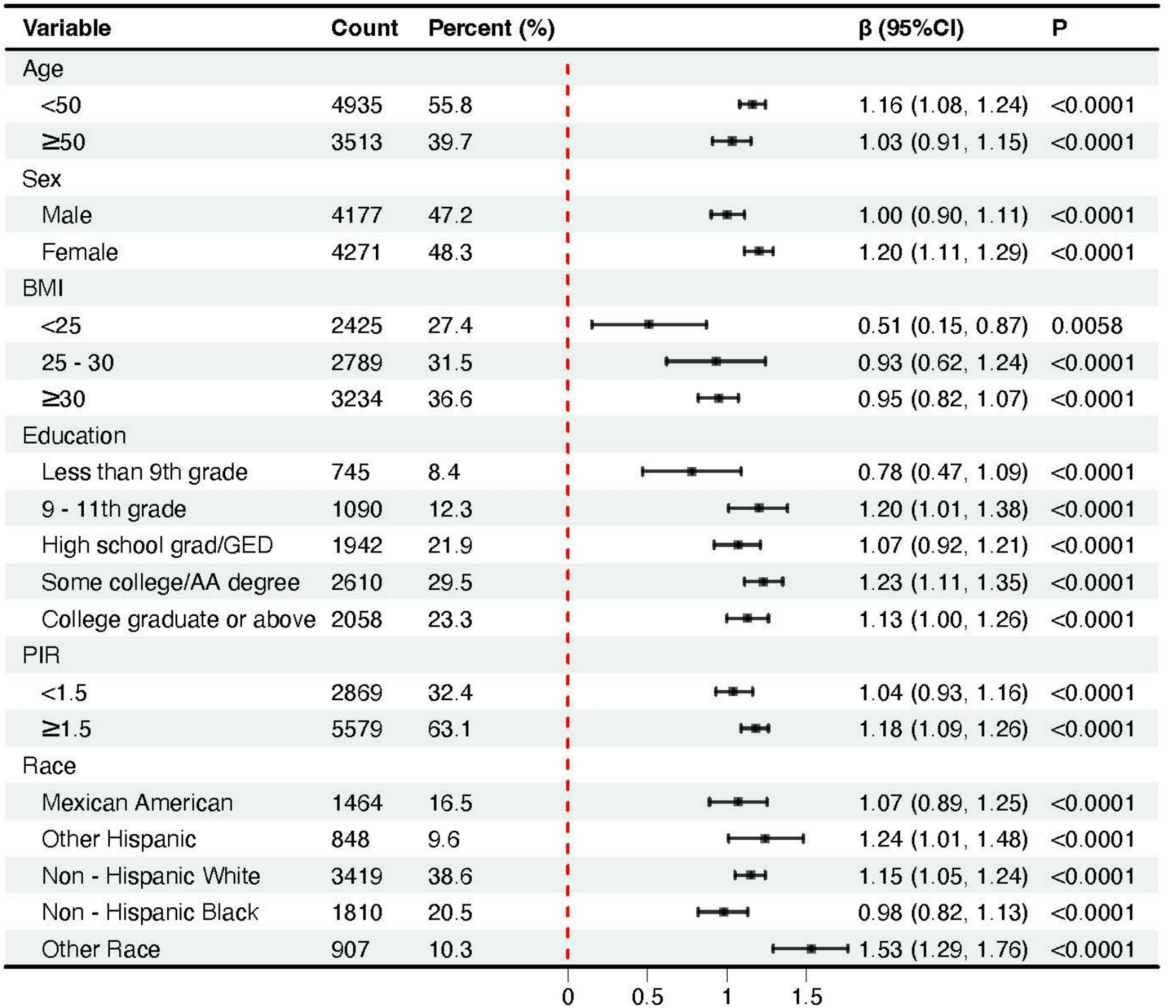
Forest plots of the association between body roundness index and phenoage acceleration across subgroups stratified by covariates.

Across all subgroups, higher BRI values were consistently associated with an increase in phenoage acceleration (Figure 3). We noticed that as many as 654 individuals had a BMI of less than 25, yet their BRI exceeded 3.78, with 55 individuals surpassing 5.01, and even one individual exceeding 6.64 (Q4). Even within the group with a BMI of less than 25, higher BRI were still associated with greater phenoage acceleration (*β =* 0.51, 95% CI: 0.15–0.87). For participants who were overweight or obese (BMI 25–30 or ≥30), the association between BRI and phenoage was more pronounced.

## Discussion

In this study, we analyzed data from the NHANES database and found a clear and significant association between the BRI and phenoage acceleration. Analysis across all models indicated that the magnitude of age acceleration (defined as the difference between phenotypic and chronological age) exhibited a positive gradient with increasing BRI levels. Accordingly, individuals with higher BRI may experience accelerated aging, potentially accompanied by an increased risk of diseases or poorer overall health outcomes. These findings highlight the importance of BRI not only as a novel body shape index but also as a sensitive predictor of aging speed, offering valuable insights into its potential clinical applications.

Obesity poses significant challenges to public health, contributing to a substantial global burden on cardiometabolic morbidity and mortality. Central adiposity, particularly visceral obesity, has emerged as a critical risk factor due to its strong association with cardiometabolic diseases and other health risks (18–20). As the prevalence of obesity continues to rise, there is an increasing need for precise and reliable methods to measure obesity to effectively predict and address these risks. Traditionally, BMI has been used to assess obesity, but it primarily reflects subcutaneous fat, which is less predictive of health outcomes than visceral fat. Individuals with the same BMI can exhibit significant differences in fat distribution and body composition (21). Additionally, a study by Rocco B. et al. found that the evaluation of central adiposity can be affected by height-standardized metrics such as BMI, unless additional indicators, such as plasma lipid concentrations, are considered (22).

The human body can be approximated as an ellipse, with the long axis representing height and the short axis representing waist circumference (17). Using this model, BRI is derived as the eccentricity of the ellipse. Thus, it is reasonable to hypothesize that BRI serves as a superior anthropometric indicator of abdominal obesity, offering a more nuanced understanding of an individual’s metabolic and physiological status. This makes BRI a more precise indicator for assessing central obesity and its associated health risks, providing improved predictive power for cardiometabolic outcomes and mortality (23, 24). The association between BRI and phenoage acceleration underscores the broader implications of body composition and fat distribution on health and aging. In the current study, the impact of BRI on phenoage is significant across all BMI subgroups, suggesting that even within the normal BMI range, an increased BRI may still contribute to accelerated aging. As body shape indicators, both BMI and BRI demonstrate a degree of mutual validation in their effects on phenoage. However, BRI is more sensitive than BMI in capturing the relationship between body composition and biological aging. This heightened sensitivity may stem from BRI’s ability to account for central adiposity, a well-established risk factor for metabolic disorders, cardiovascular diseases, and other age-related conditions. As such, BRI provides a more comprehensive assessment of an individual’s health and aging trajectory.

Accelerated biological aging, indicated by a larger discrepancy between biological age (e.g., phenoage) and chronological age, is strongly associated with adverse health outcomes (4, 25). It signifies the cumulative effects of physiological wear and tear, leading to increased vulnerability to chronic diseases such as cardiovascular disease, diabetes, and cancer (26, 27). This accelerated aging process is also linked to systemic inflammation, immune dysfunction, and reduced organ function, all of which contribute to higher mortality risk (28). Furthermore, it serves as a robust predictor of health decline, including frailty, cognitive impairment, and diminished physical performance, allowing for early identification of at-risk individuals and the implementation of preventive measures to mitigate health deterioration. Our study suggests that individuals with higher BRI may be at greater risk of adverse outcomes. Previous studies have demonstrated a significant association between elevated BRI and an increased risk of cardiovascular and metabolic disorders, as well as cancer (12, 29, 30). These conditions may contribute to accelerated aging, which in turn creates a positive feedback loop that further heightens the risk of cardiovascular and other diseases. This also helps explain the positive correlation between BRI and all-cause mortality (16). These findings underscore the potential of BRI as a predictive tool for identifying individuals at risk of accelerated aging and associated health complications. By integrating BRI into clinical practice, patients could be more effectively stratified based on their aging risk, allowing for targeted interventions to mitigate these risks.

Phenoage, a widely used biological aging metric, includes biomarkers like CRP, which is closely linked to adiposity, inflammation, and metabolic health (31, 32). As both a mediator and confounder, CRP may partially influence the observed relationship between higher BRI values and phenoage acceleration, which may indicate a potential limitation of the current BRI formula. Alternative aging measures that exclude CRP, such as epigenetic clocks, have been proposed. While highly precise, epigenetic clocks require specialized techniques, which limit their feasibility for large-scale applications (33). These limitations emphasize the need for more comprehensive and accessible biological aging metrics. Future research should explore diverse measures and account for shared pathways, such as inflammation and metabolic dysfunction, to improve accuracy and applicability.

Furthermore, the use of BRI as a predictor of aging also holds profound implications for clinical interventions. BRI could be used to guide strategies aimed at delaying aging, such as lifestyle modifications, weight management, and other preventative measures. In addition, BRI could play a critical role in evaluating the efficacy of anti-aging interventions by providing a measurable and sensitive indicator of changes in biological aging. The clinical application of BRI in predicting and managing aging-related health risks is particularly promising. Unlike BMI, which has long been criticized for its inability to differentiate between fat and lean mass or to account for fat distribution (34), BRI offers a more precise and reliable measure of body shape and composition. This makes it a valuable addition to the toolkit of clinicians and researchers working to understand and address the complex interplay between body composition, aging, and health. However, the BRI quartile thresholds were derived from the NHANES population distribution, which ensures internal consistency but also underscores the need for standardized clinical cutoffs to fully realize the translational potential of BRI across diverse populations.

Certain limitations of this study should be acknowledged. First, the declining response rate in the NHANES database may introduce non-response bias (16). Second, due to the absence of certain laboratory data, such as CRP, we were unable to include data from 2011 to 2014 period, resulting in a relatively smaller sample size. Furthermore, the participants in the NHANES database are primarily from the U. S. population, and differences in fat distribution across regions and ethnicities may exist. Therefore, future research would benefit from a larger sample size and the inclusion of participants from more diverse regions and ethnic backgrounds to ensure the validity and accuracy of these findings when applied to promoting global health.

## Conclusion

In conclusion, our study highlights the significant positive relationship between BRI and phenoage acceleration, emphasizing the potential of BRI as a sensitive and practical predictor of biological aging. Incorporation of BRI into routine assessments is anticipated to facilitate more personalized and effective approaches for promoting healthy aging.

## Data Availability

Publicly available datasets were analyzed in this study. This data can be found at: the data are publicly available on the NHANES website: https://wwwn.cdc.gov/nchs/nhanes/.
