# Supplementary figures and images for: Association between body roundness index and phenoage acceleration among US adults
Source: Front Public Health. 2025 Jun 17;13:1592274. doi: 10.3389/fpubh.2025.1592274 (PMC12209376; doi:10.3389/fpubh.2025.1592274)

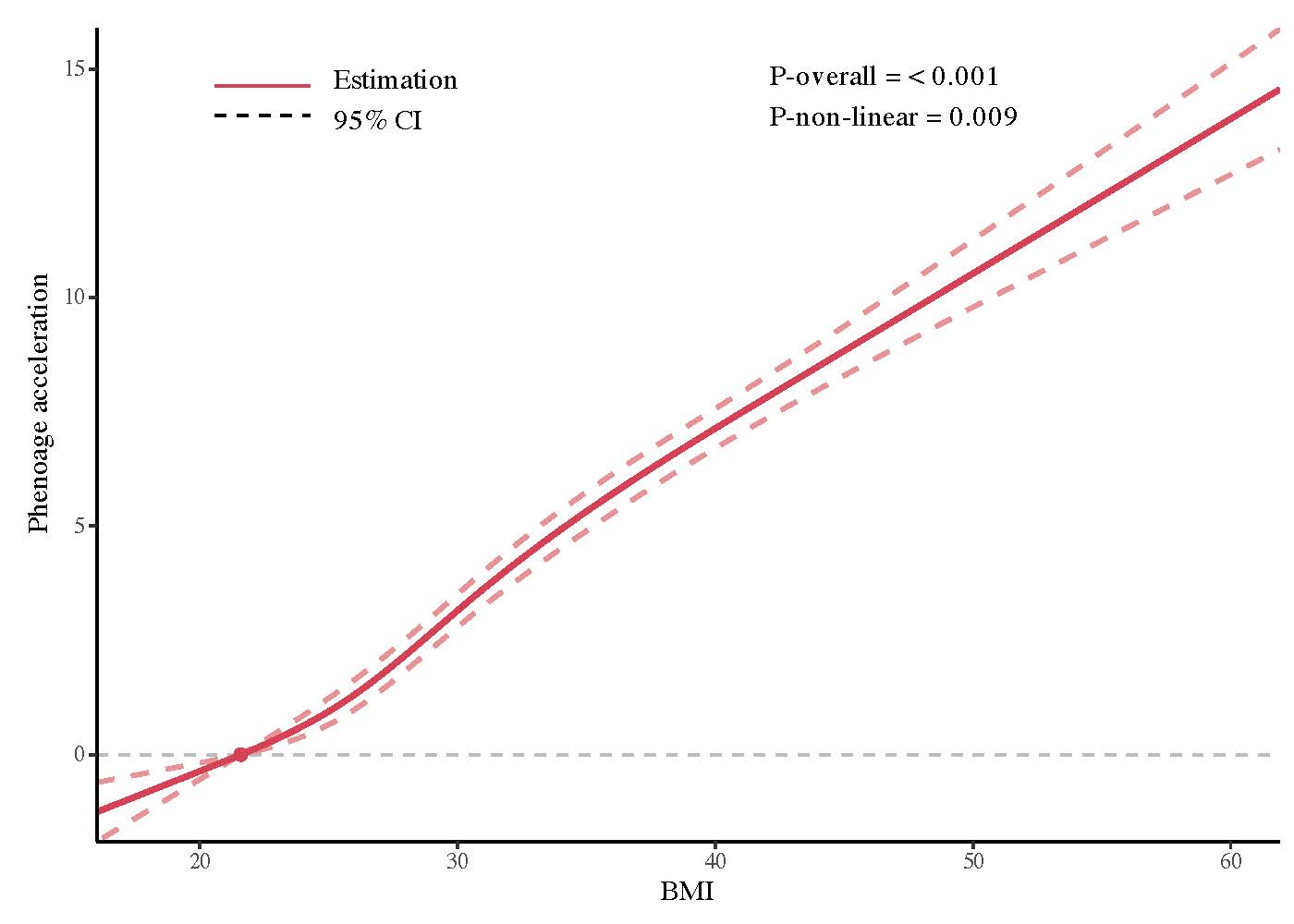

Supplement: Supplementary file 1 [file Image_1.jpg]
