# Supplementary material for: Association between body roundness index and phenoage acceleration among US adults
Source: Front Public Health. 2025 Jun 17;13:1592274. doi: 10.3389/fpubh.2025.1592274 (PMC12209376; doi:10.3389/fpubh.2025.1592274)
Supplement: Supplementary file 2 [file Supplementary_file_1.docx]

**Table S1** Association between body mass index and phenoage acceleration according to multivariate regression models

| Without adjustment | *β* (95%CI) | *P* |
| --- | --- | --- |
| Q1 (<24.4) | Reference |  |
| Q2 (24.4-28.1) | 1.54 (1.07-2.00) | <0.0001 |
| Q3 (28.1-32.9) | 3.42 (2.95-3.88) | <0.0001 |
| Q4 ($>$32.9) | 7.35 (6.89-7.82) | <0.0001 |
| Adjusted for demo information |  |  |
| Q1 (<24.4) | Reference |  |
| Q2 (24.4-28.1) | 1.33 (1.78-0.87) | <0.0001 |
| Q3 (28.1-32.9) | 3.07 (3.53-2.61) | <0.0001 |
| Q4 ($>$32.9) | 6.99 (7.45-6.53) | <0.0001 |
| With full adjustments |  |  |
| Q1 (<24.4) | Reference |  |
| Q2 (24.4-28.1) | 1.44 (1.87-1.01) | <0.0001 |
| Q3 (28.1-32.9) | 2.93 (3.37-2.50) | <0.0001 |
| Q4 ($>$32.9) | 6.06 (6.51-5.62) | <0.0001 |
